# IFN-γ in ovarian tumor microenvironment upregulates HLA-E expression and predicts a poor prognosis

**DOI:** 10.1186/s13048-023-01286-z

**Published:** 2023-11-25

**Authors:** Hui Zheng, Xiaolin Guan, Xin Meng, Ying Tong, Yanchun Wang, Suhong Xie, Lin Guo, Renquan Lu

**Affiliations:** 1 Department of Clinical Laboratory, Fudan University Shanghai Cancer Center, Shanghai, 200032 China; 2 Department of Oncology, Shanghai Medical College of Fudan University, Shanghai, 200032 China; 3 Institutes of Biomedical Sciences, Shanghai Medical College of Fudan University, Shanghai, 200032 China

**Keywords:** Human leukocyte antigen-E, Natural killer cell, Ovarian cancer, Tumor microenvironment

## Abstract

**Background:**

Inflammation and immunity are two main characteristics of tumor microenvironment (TME). Interferon-gamma (IFN-γ) is generally considered as a pro-inflammatory cytokine which mediates anti-tumor immune response. Recently, IFN-γ was also reported to play a protumorigenic role. However, the mechanisms of tumor-promoting effect induced by IFN-γ remain unclear.

**Methods:**

The expression of leukocyte antigen-E (HLA-E), IFN-γ, CD3 and CD56 in clinical samples of ovarian cancer was detected by mutiplexed immunohistochemistry. The mechanism to induce HLA-E overexpression by IFN-γ was explored using human ovarian cancer cell lines through western blot and flow cytometry. We further clarify the role of overexpressed-HLA-E on natural killer (NK)-mediated cell lysis.

**Results:**

We found that IFN-γ could upregulate HLA-E protein expression through activating of JAK/STAT1 signaling pathway, and increase cell surface HLA-E level through enhancing proteasome activity. We also observed that only high levels of membrane HLA-E expression contributed to the inhibition of NK-mediated cytotoxicity. We showed that progression-free survival (PFS) of ovarian cancer patients was negatively correlated with IFN-γ expression in their tumor tissues, due to more tumor infiltrating NK cells compared with T lymphocytes.

**Conclusions:**

Our study revealed the protumorigenic role of IFN-γ by upregulation of HLA-E expression and rendering tumors less susceptible to immune attack. We also provided a novel insight into the relationship between tumor microenvironment and immune evasion.

**Supplementary Information:**

The online version contains supplementary material available at 10.1186/s13048-023-01286-z.

## Introduction

A variety of components in solid tumors form a special microenvironment which affects the biological functions of tumor or immune cells within. Immunosuppressive environment is an important factor hindering tumor treatment, therefore, it is essential to clarify the mechanism of immunosuppression production [1, 2]. A large number of immune/inflammatory cells as well as cytokines are present in the tumor microenvironment (TME). It has been reported that cytokines can change the function of antigen presenting cells and lymphocytes, thereby affecting anti-tumor immune responses [3, 4]. Inflammation and immunity are two main characteristics of TME. Interferon-gamma (IFN-γ), classically considered as a pro-inflammatory cytokine, is expected to work as an anti-tumor agent [5, 6]. IFN-γ-mediated immunity was used in many clinical trials to treat with different types of cancer [7]. However, the outcomes were not satisfactory with limited success. Even worse, tumor progressed after administration of IFN-γ to ovarian cancer patients [8]. Some studies suggested that IFN-γ perhaps played a protumorigenic role that was used as an inducer to inhibit anti-tumor immune response by malignant cells [9, 10]. The mechanisms about tumor-promoting effect of IFN-γ remain to be explored.

Human leukocyte antigen-E (HLA-E) is a non-classical MHC molecule whose overexpression in several human cancer types has been reported [11–13]. Our previous study also indicated that HLA-E protein was overexpressed in ovarian cancer tissues compared with normal ovary tissues [14]. *HLA-E* gene is ubiquitously transcribed in all human tissues. The differential expression of HLA-E protein between ovarian cancer and normal tissues may be controlled by post-transcriptional regulation [15]. However, how to produce the different regulation remains unknown. More interestingly, not all tumor cell lines overexpressed HLA-E, even without HLA-E expression, which is inconsistence of cancer tissues [16]. Thus, HLA-E expression in cancer tissues may be regulated by TME.

In this paper, the expression of IFN-γ and HLA-E was detected in ovarian cancer tissues. The mechanism of IFN-γ-induced HLA-E upregulation was investigated using ovarian cancer cell lines. The inhibitory effect on natural killer (NK) cells attack by upregulation of HLA-E was demonstrated. We also analyzed the relationship between IFN-γ and progression-free survival (PFS) in ovarian cancer patients.

## Results

### IFN-γ is positively correlated with HLA-E protein expression in ovarian cancer tissues

**Fig. 1:**
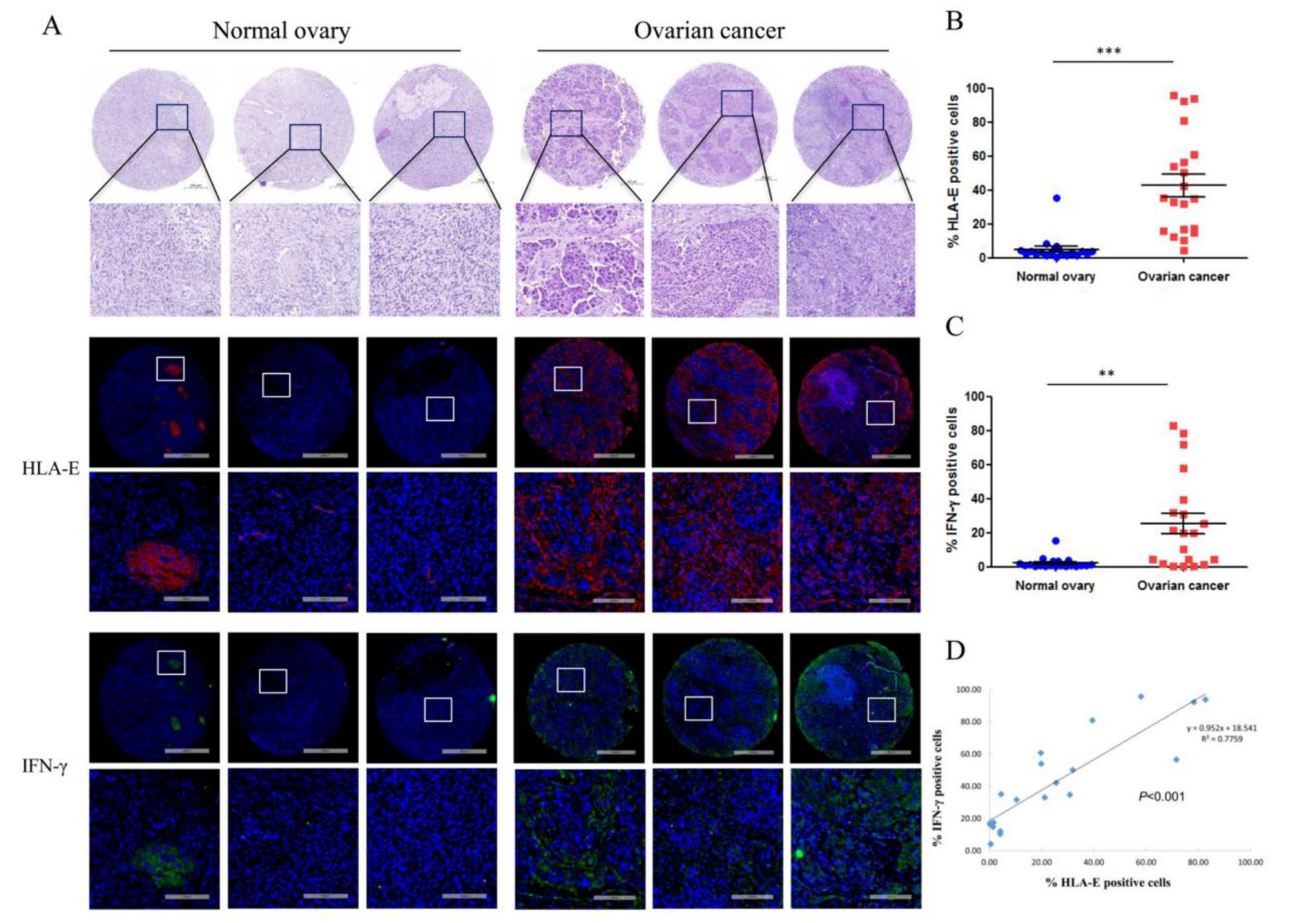
The expression of HLA-E and IFN-γ in normal or ovarian cancer tissues. (**A**) Three representative pictures of HLA-E and IFN-γ expression in normal or ovarian cancer tissues with HE staining. HLA-E: red fluorescence, IFN-γ: green fluorescence. (**B**) The statistics of HLA-E-positive cells in 20 normal and 20 ovarian cancer tissues. ^***^P < 0.001. (**C**) The statistics of IFN-γ-positive cells in 20 normal and 20 ovarian cancer tissues. ^**^P < 0.01. (**D**) The relationship between HLA-E and IFN-γ expression in ovarian cancer tissues

HLA-E and IFN-γ expression in tissue microarray with 20 normal ovary tissues and 20 ovarian cancer tissues was detected by multiplexed immunohistochemistry. The representative images in Fig. 1A showed a negative or weak expression of HLA-E and IFN-γ in normal ovary, a moderate or strong expression of HLA-E and IFN-γ in cancer samples, and HLA-E expression was consistent with our previous results [14]. The percentage of HLA-E or IFN-γ positive cells in ovarian cancer tissues was significantly higher than in controls (Fig. 1B, C). There was a positive correlation between HLA-E and IFN-γ expression in ovarian cancer tissues (Fig. 1D). The whole image of tissue microarray was available in supplemental section (Additional file 2).

### IFN-γ induces HLA-E expression in ovarian cancer cells

**Fig. 2:**
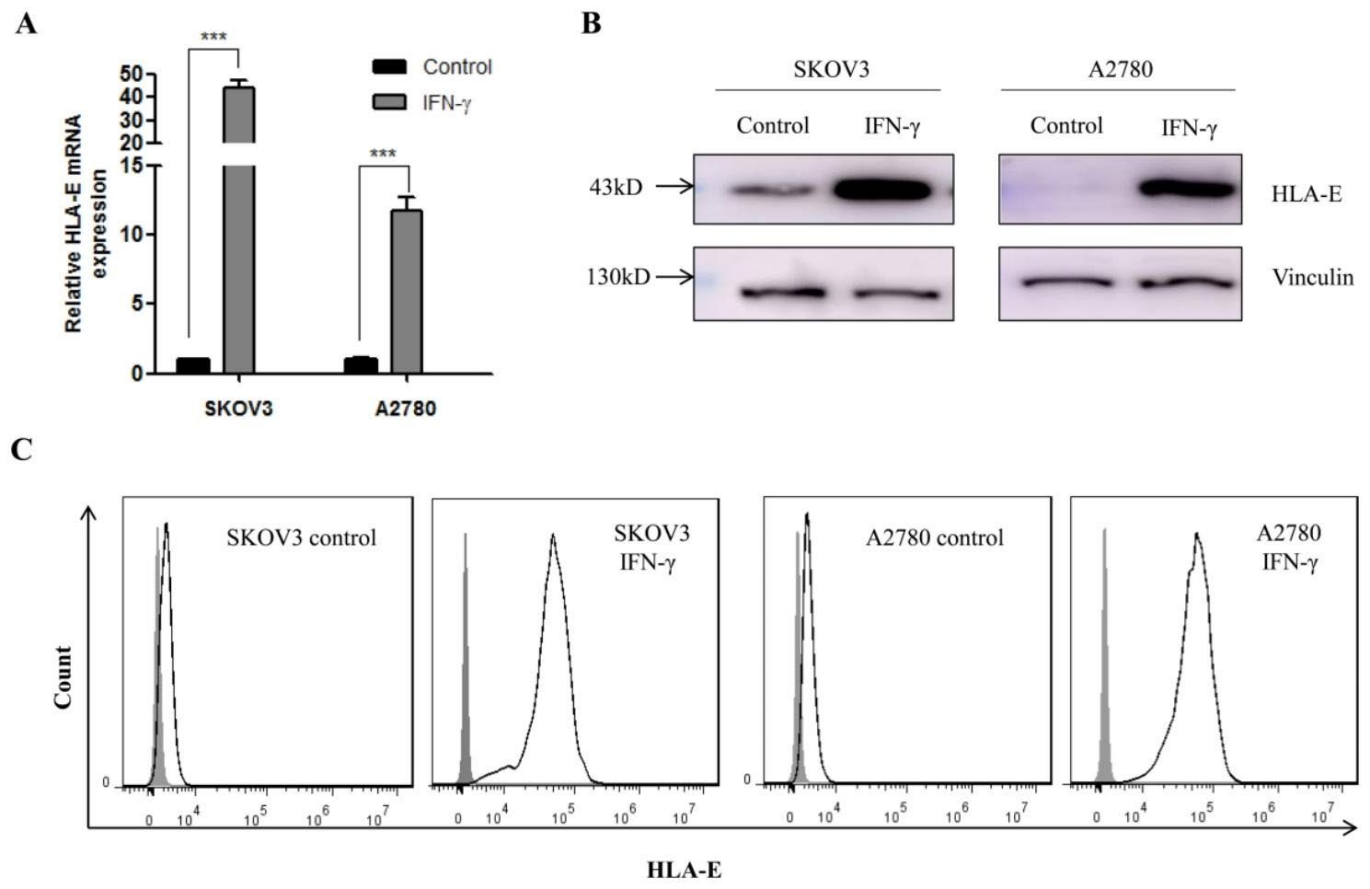
HLA-E expression in tumor cells after treatment with IFN-γ in vitro. IFN-γ-induced HLA-E upregulation was demonstrated by quantitative real-time PCR (**A**), western blot (**B**), and flow cytometry(**C**). IFN-γ: the cells were treated with 25ng/mL recombinant human IFN-γ for 24 h; Control: the cells were cultured without IFN-γ adding. Open histogram: anti-HLA-E antibody; filled histogram: isotype control. ^***^P < 0.001. Data shown represented one of three independent experiments

To further verify HLA-E expression of tumor cells is induced by IFN-γ in TME, two human ovarian cancer cell lines SKOV3 (high HLA-E protein expression) and A2780 (low HLA-E protein expression) were used for further investigation. Recombinant human IFN-γ was added into cell culture medium to treat for 24 h, HLA-E situation was detected through real-time quantity PCR, western blot and flow cytometry. We found that HLA-E mRNA and protein expression was significantly upregulated in two ovarian cancer cell lines (Fig. 2A, B). Furthermore, cell surface HLA-E expression was also obviously induced (Fig. 2C). Hence, the expression of HLA-E was inducible in tumor cells.

### Suppressing STAT1 signaling reduces HLA-E protein expression but has no effect on NK lysis

**Fig. 3:**
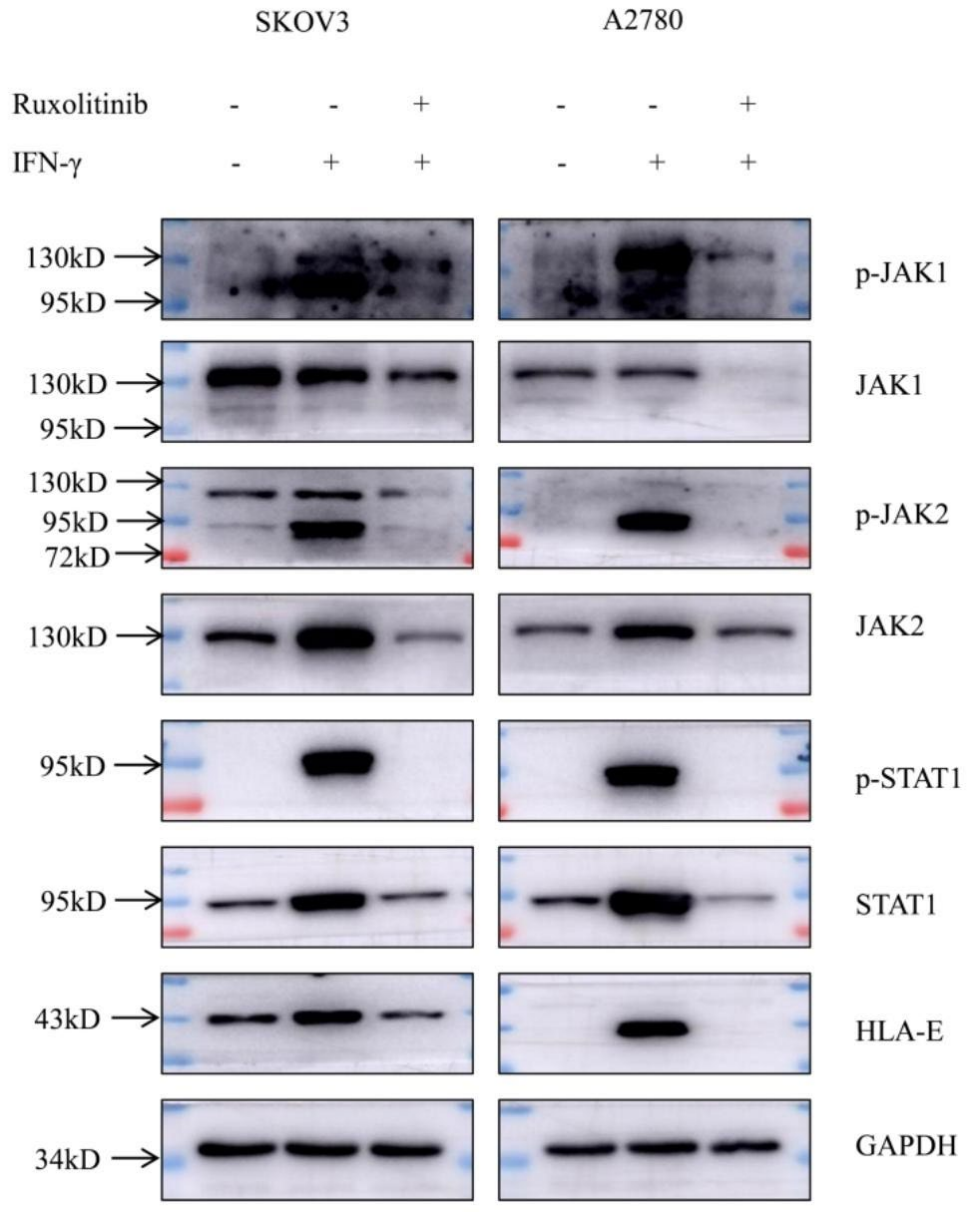
IFN-γ-induced HLA-E overexpression was associated with JAK/STAT1 signaling pathway. IFN-γ treatment activated JAK1/2 and STAT1, and HLA-E expression was not upregulated by IFN-γ in the presence of JAK1/2 inhibitor (50µM Ruxolitinib). Data shown represented one of three independent experiments

**Fig. 4:**
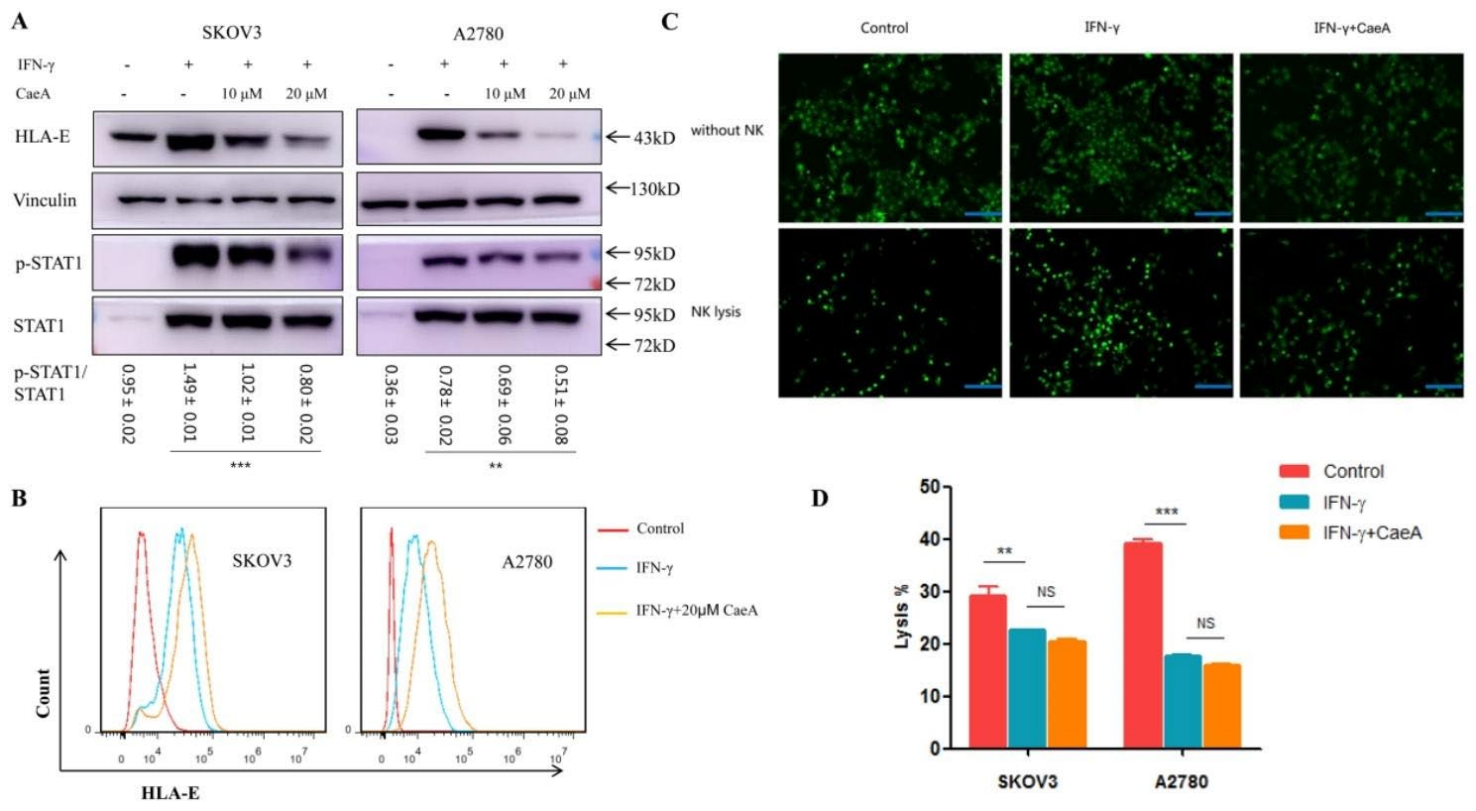
IFN-γ upregulated HLA-E expression through activation of STAT1 signal transduction pathway. (**A**) Caerulomycin A suppressed IFN-γ-induced STAT1 phosphorylation. HLA-E protein expression was also decreased with the increase of Caerulomycin A concentration. (**B**) Cell surface HLA-E expression was not downregulated by Caerulomycin A treatment. (**C**) SKOV3 cells transfected with EGFP were observed after being cultured with NKL cells for 4 h. More tumor cells were survived in IFN-γ stimulating group than in control group (without treatment). Caerulomycin A did not abrogate the IFN-γ-induced survivals. The bars = 250 μm. (**D**) IFN-γ reduced the function of NK lysis, and Caerulomycin A had no effect on IFN-γ-mediated killing inhibition, which was shown through LDH cytotoxicity assay kit. ^**^P < 0.01, ^***^P < 0.001. Data shown represented one of three independent experiments

Previous work by others showed that transcriptional regulation of HLA-E was mediated by STAT1 binding site [17]. Therefore, we clarified the mechanism of IFN-γ-induced HLA-E overexpression focusing on JAK/STAT1 signaling pathway. We indicated that IFN-γ treatment could phosphorylate JAK1/2 and STAT1, meanwhile, increase total JAK2 and STAT1 expression. Ruxolitinib, a selective inhibitor of JAK1/2, blocked JAK1/2 expression and phosphorylation significantly. As a downstream of JAK pathway, STAT1 was also influenced by Ruxolitinib. HLA-E protein in ovarian cancer cells could not be upregulated by IFN-γ in the presence of Ruxolitinib (Fig. 3). To demonstrate the regulation depending on STAT1 signaling, we used Caerulomycin A (CaeA) to specifically suppress STAT1 activation. We showed that CaeA could reduce the IFN-γ-induced STAT1 phosphorylation, and HLA-E protein level was gradually decreased with increasing CaeA concentration (Fig. 4A). However, surface HLA-E expression was not downregulated by CaeA treatment (Fig. 4B). In most cases, HLA-E presents peptides to NK cells by interacting with its inhibitory or stimulatory receptors [18]. NK lysis assay was performed using NKL cell line containing many receptors, and investigated by fluorescence microscope and LDH release method. IFN-γ-induced overexpression of HLA-E could protect ovarian cancer cells from NK lysis. Suppressing STAT1 signaling reduced HLA-E protein expression but did not abrogate HLA-E-mediated NK inhibition (Fig. 4C, D).

### IFN-γ inhibits NK lysis depending on high levels of surface HLA-E expression modulated by proteasome activity

**Fig. 5:**
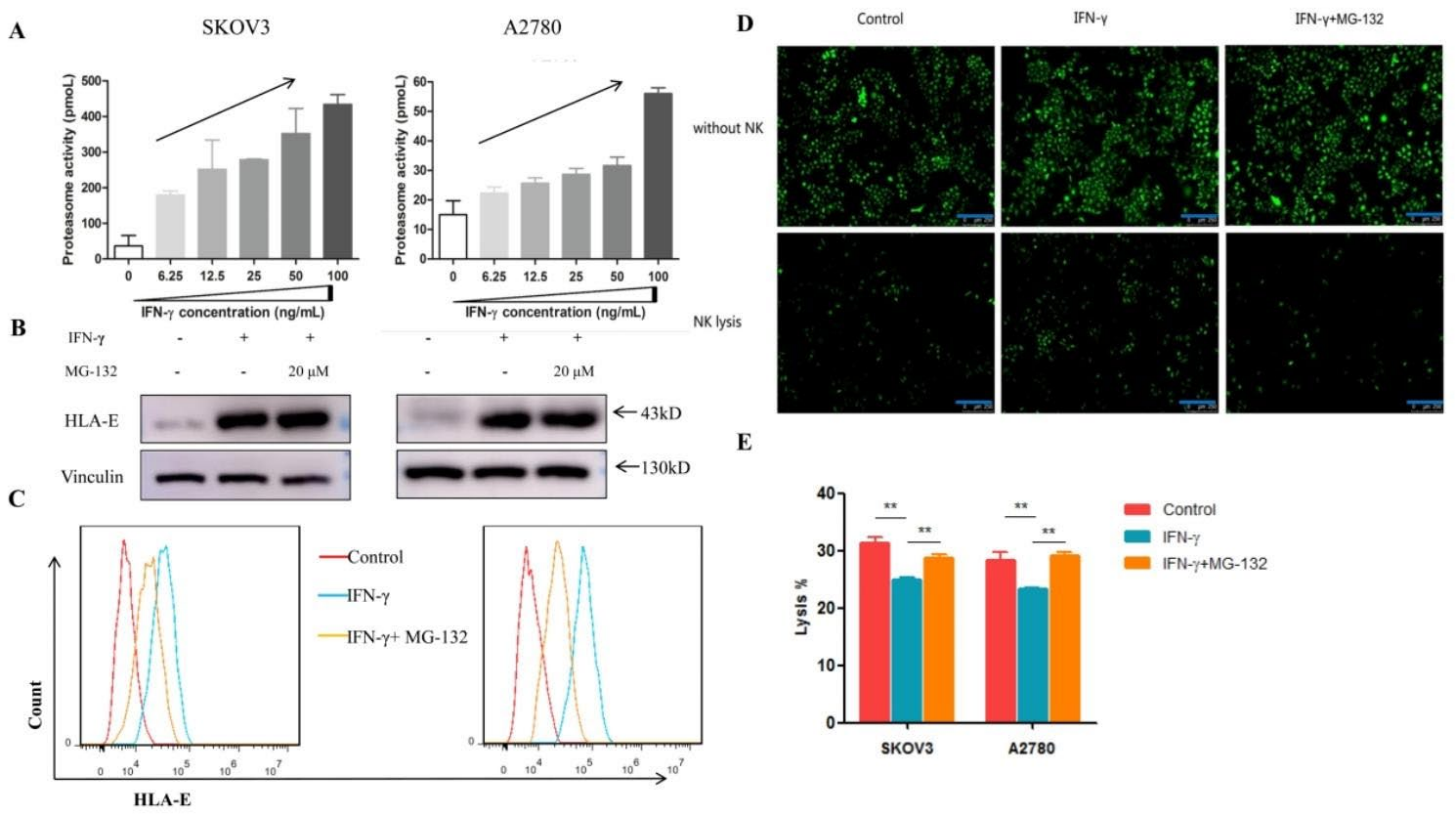
IFN-γ-mediated inhibition of NK lysis was modulated by proteasome activity. (**A**) The ovarian cancer cells were treated with gradient concentration of IFN-γ for 24 h. IFN-γ could increase proteasome activity of the tumor cells. (**B**) Proteasome inhibitor MG-132 had no influences on IFN-γ-induced upregulation of total HLA-E protein. (**C**) Surface HLA-E expression was reduced with MG-132 treatment. (**D**) SKOV3 cells transfected with EGFP was observed after being cultured with NKL cells for 4 h. More tumor cells were survived in IFN-γ stimulating group than in control group. MG-132 could reduce the survival rate to abrogate the effect of IFN-γ. The bars = 250 μm. (**E**) LDH cytotoxicity assay was used to indicate the function of NK lysis. IFN-γ reduced the cytotoxicity of NK cells significantly compared with control and MG-132 treatment groups. ^**^P < 0.01. Data shown represented one of three independent experiments

The effect of HLA-E on NK cells seems to be dependent on surface HLA-E expression levels. Endogenous antigen processing and presentation through proteasome pathway is required for the stable expression of HLA-E on cell surface. Under inflammatory conditions, the constitutively expressed immunoproteasome subunits are replaced by those with catalytic activity [19]. We further demonstrated that IFN-γ could increase proteasome activity of tumor cells (Fig. 5A). MG-132, blocking the proteolytic activity of the 26S proteasome complex, did not affect total HLA-E protein levels in ovarian cancer cells, while effectively downregulating surface HLA-E expression (Fig. 5B, C). Thereafter, MG-132 could make tumor cells re-susceptible to NK lysis (Fig. 5D, E).

### High level of IFN-γ predicts poor prognosis in ovarian cancer patients

**Fig. 6:**
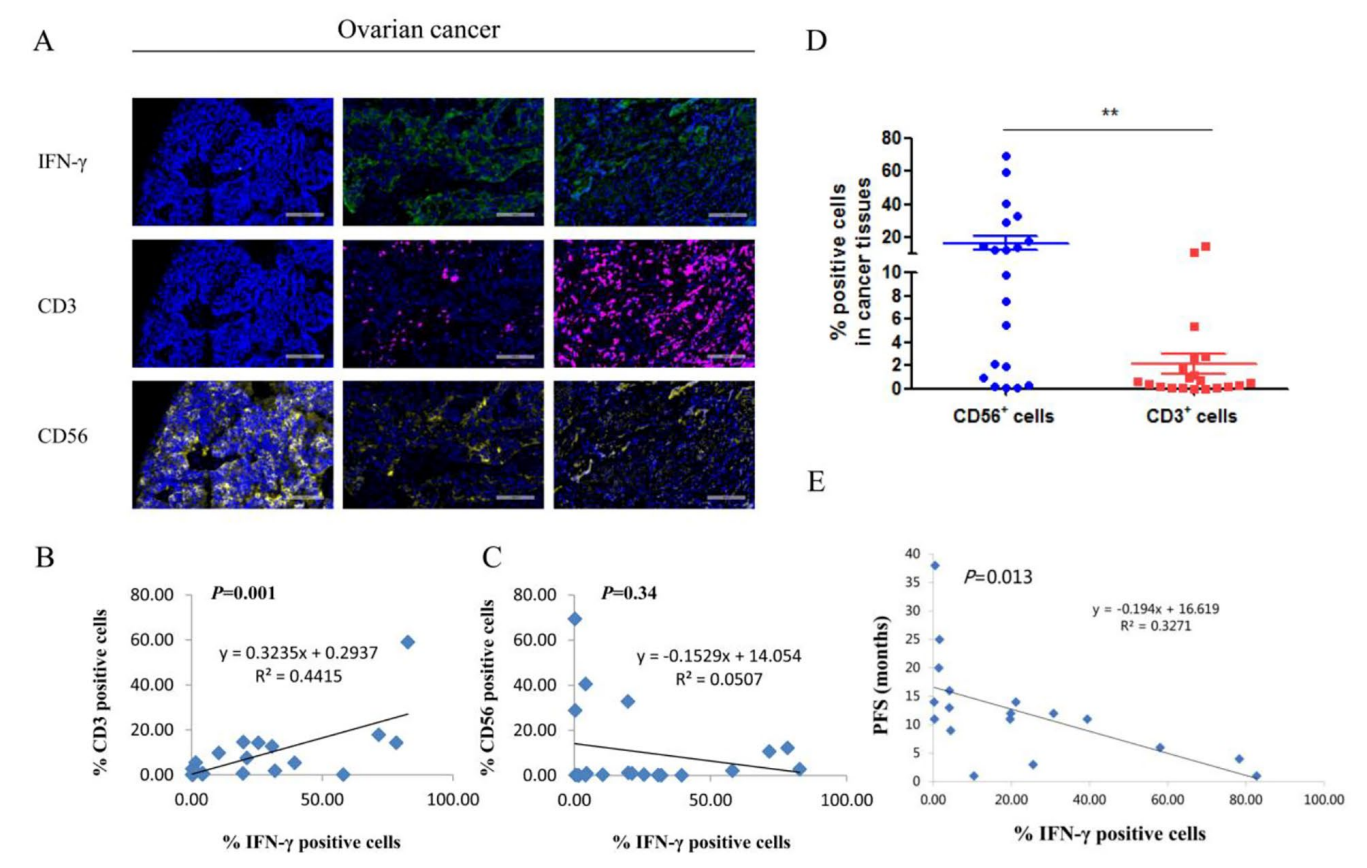
(**A**) Three representative pictures of IFN-γ, CD3 and CD56 expression in ovarian cancer tissues. IFN-γ: green fluorescence, CD3: purple fluorescence, CD56: yellow fluorescence. (**B**) The relationship between IFN-γ and CD3 expression in ovarian cancer tissues. (**C**) The relationship between IFN-γ and CD56 expression in ovarian cancer tissues. (**D**) The percentage of CD3 and CD56 positive cells in 20 ovarian cancer tissues. ^**^P < 0.01. (**E**) The relationship between IFN-γ and PFS in ovarian cancer patients

IFN-γ is a cytokine that is primarily secreted by immune cells in TME. The co-staining with IFN-γ, CD3 and CD56 antibodies by multiplexed immunohistochemistry suggested that IFN-γ secretion was positively correlated with CD3^+^ cells infiltrating (Fig. 6A, B). The percentage of CD56^+^ cells was not related to IFN-γ (Fig. 6A, C). Therefore, in our tested cases, IFN-γ is primarily secreted by tumor infiltrating T cells. Accordingly, there was a weak expression of CD3 in normal ovary tissues (Additional file 2). IFN-γ then upregulates HLA-E expression in ovarian cancer cells, which presents peptides to inhibitory receptors on NK cells. We noticed more CD56^+^ cells infiltrating in ovarian cancer tissues compared with CD3^+^ cells (Fig. 6D), which most likely neutralized the anti-tumor effect by IFN-γ-induced T cell activation. The twenty ovarian cancer patients analyzed above were followed up (two missing cases) and showed a weak negative correlation between IFN-γ expression and PFS (Fig. 6E).

## Discussion

Ovarian cancer has the highest mortality among gynecological malignant tumors. New treatment strategies are of great need for ovarian cancer patients. With the improved understanding of immune recognition and regulation of cancer cells, cancer immunotherapy has attracted significant interest. However, the immunosuppressive tumor microenvironment has become a major barrier to successful cancer immunotherapy for ovarian cancer patients [20]. The molecules within it have not been investigated clearly. HLA-E is significantly upregulated in ovarian cancer tissues, and HLA-E presents peptides to inhibitory receptor CD94/NKG2A or stimulatory receptor CD94/NKG2C expressing on the majority of NK cells. The affinity of HLA-E for CD94/NKG2A is about eight times higher than for CD94/NKG2C [21]. Therefore, the interaction between surface HLA-E on tumor cells and NK receptors usually leads to tumor escape from NK lysis. Further investigations showed distinct HLA-E/peptide complexes would result in different effects to NK cells. The peptides derived from various HLA class I leader sequences block conventional NK cells with CD94/NKG2A receptor, while HLA-G leader peptides stimulate adaptive NK cells expressing CD94/NKG2C receptor [22]. Although HLA-G was expressed in various types of tumors [23], many unknown factors in TME will influence the interaction between HLA-E and CD94/NKG2 receptors. In present study, we showed that IFN-γ-induced HLA-E upregulation would decrease NK lysis to tumor cells. We did not explore the exact ligand of NK cells associated with functional assays, however, the results supported that surface HLA-E on tumor cells primarily recognized inhibitory receptors on NK cells.

Pro-inflammatory factor IFN-γ was secreted in TME, and was reported to induce PD-L1 expression in ovarian cancer cases [24]. PD-L1 is the ligand of PD-1 on lymphocytes, their interaction will inhibit lymphocyte activation. So the function of IFN-γ to immune response is complex. Besides the classical biological function to promote cellular immunity, the immune suppressive of IFN-γ needs to be considered. In the present study, we found a positive correlation between IFN-γ and HLA-E expression in ovarian cancer tissues. Using ovarian cancer cell lines, the modulation role of IFN-γ was verified. We indicated that IFN-γ could increase intracellular HLA-E protein level through activation of JAK/STAT1 signaling, but the HLA-E membrane expression was not regulated by this pathway. Consistent with results as we known, the cell surface HLA-E was related to antigen processing and presentation pathway. We suggested that IFN-γ induced HLA-E membrane expression through proteasome pathway, and which would inhibit NK attack. The experimental system with tumor cell lines cannot completely replace the clinical reality. HLA-E expression was regulated by many factors in TME. We also found TNF-α could upregulate HLA-E protein expression in tumor cells (data was not shown). However, through cell experiments in vitro, we could clarify the definite role of IFN-γ without interfering by other factors. We think our results should be one of the real mechanisms of HLA-E overexpression in vivo. Furthermore, we also showed a weak negative correlation between IFN-γ and PFS. Paradoxically, tumor cells also can take advantage of IFN-γ cytokine to make target cells more susceptible to T lymphocytes. IFN-γ seems to have two faces, immunoactivating/immunoregulatory and antitumor/tumor-promoting [25]. In the clinical samples of ovarian cancer, we found more tumor infiltrating NK cells compared to T lymphocytes, which maybe an explain for the poor prognosis contributed by IFN-γ in TME. Thus, IFN-γ treatment in ovarian cancer should be considered the alternative action of tumor promotion. Most recent, the HLA-E-NKG2A axis was regarded as a novel checkpoint in TME [26]. We supposed that IFN-γ conjunction with HLA-E-NKG2A inhibitor would suppress the ‘bad side’ of IFN-γ and produce good prognosis.

## Conclusions

We have shown in this study that IFN-γ within TME predicted a poor prognosis through upregulation of HLA-E expression, especially the cell membrane HLA-E expression with increasing proteasome activity. The high levels of cell surface HLA-E expression contributed to the tumor escape from immune surveillance, through inhibiting NK lysis. We provided a novel insight into the relationship between tumor microenvironment and immune evasion.

## Materials and methods

### Cell lines and treatment

Two human ovarian cancer cell lines (SKOV3 and A2780) were originally obtained from ATCC and kept in our lab. The cell lines were grown at 37^o^C in a humid atmosphere containing 5% CO_2_ with RPMI 1640 medium, supplemented with 10% fetal calf serum and 100U/mL penicillin plus 100 g/mL streptomycin (all from gibco). Cytokines were purchased from PeproTech. To test the regulation role of IFN-γ to HLA-E expression, the tumor cells were seeded into 6-well plate (2 × 10^5^/well) for 24 h, then treated with 25ng/mL recombinant human IFN-γ for another 24 h. To explore the specific mechanisms of IFN-γ regulation, IFN-γ-treated tumor cells were further cultured with or without Caerulomycin A (MCE, HY-114,495) for 48 h, or MG-132 (MCE, HY-13,259) for 6 h. To completely block JAK signaling pathway, cells were pretreated with 50µM Ruxolitinib (Selleck, S1378) for 1 h, followed by IFN-γ stimulating for 24 h in the presence of the inhibitor.

### Detection of HLA-E expression by real-time PCR

Cell RNA was extracted using the RNA-Quick purification kit (ES Science). Up to 1 µg total RNA was reverse transcribed to cDNA by PrimeScript RT reagent kit (Takara). The primers were as follows: HLA-E forward: 5’-CCG TCA CCC TGA GAT GGA AG-3’, HLA-E reverse: 5’-ACA GCT CCA GAG ACC ACA GA-3’; β-actin forward: 5’-ACA CTG TGC CCA TCT ACG AGG-3’, β-actin reverse: 5’-AGG GGC CGG ACT CGT CAT ACT-3’. Quantitative PCR assay was performed using Hieff qPCR SYBR Green Master Mix (YEASEN) and Applied Biosystems QuantStudio Dx system. All PCR reactions were performed in triplicate. The relative expression value for each sample was shown as 2^−ΔΔCt^.

### Western blot analysis

Homogenization of cells was carried out in cell lysis buffer containing PMSF and protease inhibitor cocktail (all from Beyotime), then proteins were separated in SDS-PAGE gel and electroblotted onto nitrocellulose membranes. The primary antibodies were used as follows: HLA-E antibody (Abcam, MEM-E/02, 1:1000), Vinculin antibody (CST, #4650, 1:500), GAPDH antibody (Proteintech, 60004-1-Ig, 1:50000), IFN-γ signaling pathway antibody sampler kit containing antibodies of JAK1, p-JAK1, JAK2, p-JAK2, STAT1, p-STAT1 (CST, #44,902, 1:1000), which were incubated at 4 ^o^C overnight. After washing, the secondary antibody conjugated to horseradish peroxidase (HRP) was incubated at room temperature for 1 h. The protein bands were detected with ELC reagent (YEASEN) on Amersham Imager 600 (GE Healthcare Life Sciences).

### Flow cytometry

3D12-APC mAb or mouse IgG1 kappa isotype control APC (eBioscience) was incubated at 4 ^o^C for 30 min. After washing twice, flow cytometry was used to detect surface HLA-E expression on ovarian cancer cells. The data were acquired on Cytoflex S (Beckman Coulter), and analyzed using FlowJo software.

### NK lysis assay

NKL cell line was purchased from ATCC. After ovarian cancer cells were treated, NKL (5 × 10^5^/well) cells were added and cultured for another 4 h. To observe the survived cells after killing, the ovarian cancer cells were transfected with EGFP gene. The real-time cell history recorder (Chinchilla life sciences) was used to survey and record changes at the same location. Meanwhile, LDH cytotoxicity assay kit (Beyotime) was used to evaluate the quantity of cells which were killed by NKL cells.

### Proteasome activity assay

The ovarian cancer cells treated with IFN-γ were homogenized in 0.5% NP-40 (Sangon Biotech). Proteasome activity assay kit (Abnova) was used according to the manufacturer’s instructions. In brief, each sample was added into two wells (one with proteasome inhibitor MG-132), then incubated in reaction buffer and AMC-tagged peptide substrate. One hour later, the highly fluorescent AMC was measured in a microplate reader (SpectraMax iD3, Molecular Devices). The RFU generated by proteasome is ΔRFU = RFU total - RFU inhibitor. Apply the ΔRFU to AMC standard curve to get proteasome activity concentration.

### Patients and tissue samples

A total of 20 ovarian cancer tissues and 20 normal ovary tissues were obtained from patients who underwent surgical resection at Fudan University Shanghai Cancer Center before receiving any other treatments. The normal ovary tissues were from patients with cervical cancer who were willing to remove ovary to avoid recurrence. Each specimen was evaluated in hematoxylin and eosin (HE)-stained sections by pathologists, and the images were available in additional file 1. All specimens were fixed in formalin and embedded in paraffin, and the tumor or normal tissues were selected to make a tissue microarray. The study’s protocol was reviewed and approved by the Ethics Committee of Fudan University Shanghai Cancer Center (certification No. 050432-4-1212B). Informed consents were obtained from each patient.

### Multiplexed immunohistochemistry

The tissue microarray was stained with four fluorescent dyes by tyramide signal amplification (TSA) kit (WiSee Biotechnology). Primary antibodies to HLA-E (Abcam, MEM-E/02), IFN-γ (R&D, #25,718), CD3 (Yuanxibio, #YX22005) and CD56 (GeneTech, #GT200502) were sequentially applied, followed by HRP-conjugated secondary antibody (Yuanxibio, #A10011-60) incubation. The slides were microwave heat-treated after each TSA operation, and then stained with DAPI after all the antibodies above being bound. The stained slides were scanned to obtain whole image with Aperio Versa 8 tissue imaging system. The total cells in each sample were identified and analyzed by Indica Halo software.

### Statistical analysis

Statistical analysis of the data was performed using SPSS version 22.0 software. The data were compared between two groups using an independent Student’s t-test. Pearson analysis was applied to evaluate the correlation between IFN-γ and HLA-E/CD3/CD56 or PFS. Differences were considered significant when *P* < 0.05.

### Electronic supplementary material

Below is the link to the electronic supplementary material.

Supplementary Material 1

Supplementary Material 2

## Data Availability

The data used and analyzed in current study are available from the corresponding author on reasonable request.

## Abbreviations

IFN-γ: Interferon-gamma
HLA-E: Human leukocyte antigen-E
NK: natural killer
PFS: progression-free survival
TME: tumor microenvironment
