# Supplementary figures and images for: IFN-γ in ovarian tumor microenvironment upregulates HLA-E expression and predicts a poor prognosis
Source: J Ovarian Res. 2023 Nov 25;16:229. doi: 10.1186/s13048-023-01286-z (PMC10675946; doi:10.1186/s13048-023-01286-z)

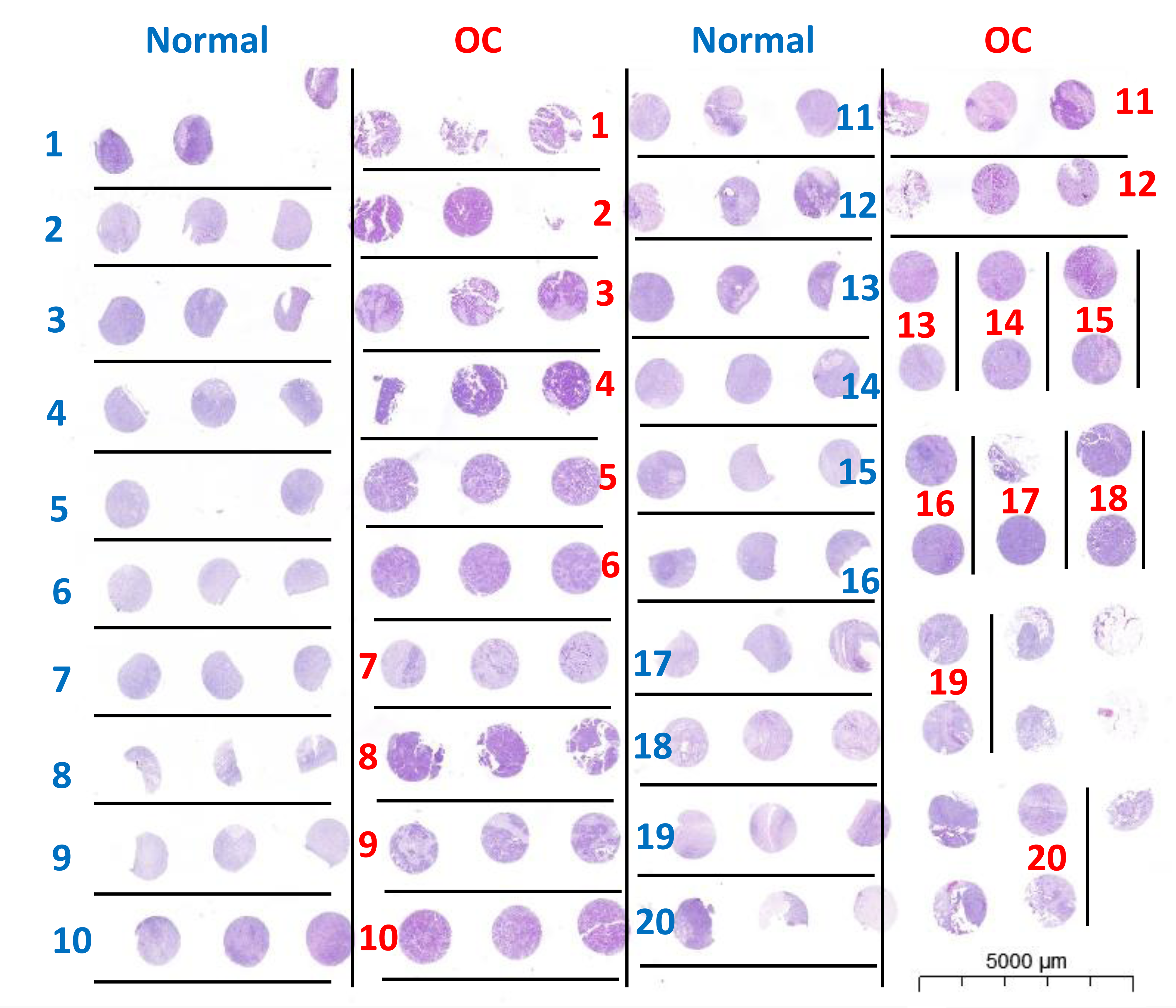

Supplement: Supplementary file 1 — Supplementary Material 1 [file 13048_2023_1286_MOESM1_ESM.png]

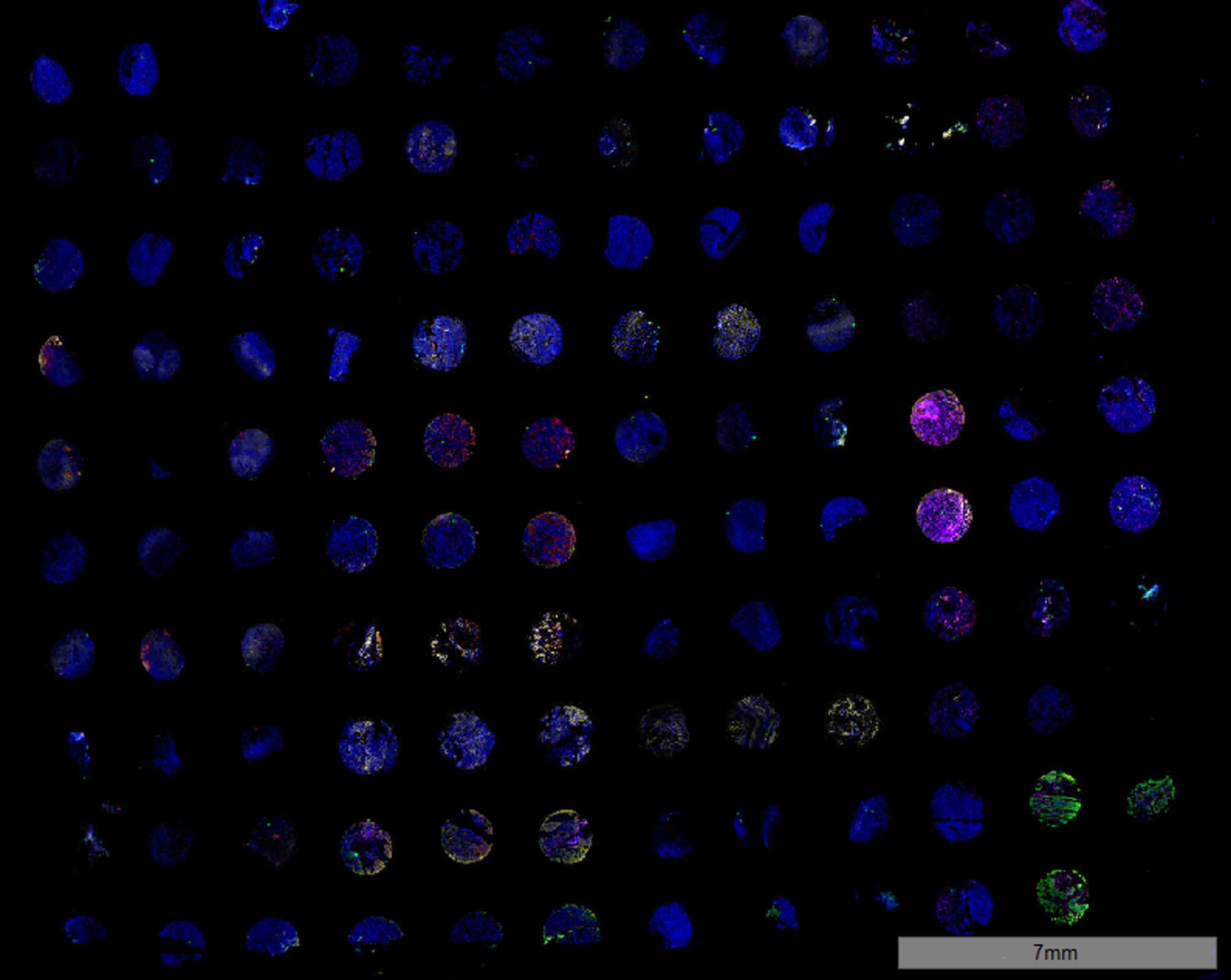

Supplement: Supplementary file 2 — Supplementary Material 2 [file 13048_2023_1286_MOESM2_ESM.png]
